# LncTRDMT1–5 promotes chromosomal instability through MSRB3-mediated cell cycle and DNA damage in breast cancer

**DOI:** 10.3389/fonc.2026.1899923

**Published:** 2026-08-05

**Authors:** Qi Chen, Yunpeng Chen, Hui Yang, Xiaolan Zhu

**Affiliations:** 1 Department of Genetic and Prenatal Diagnosis, Fourth Affiliated Hospital of Jiangsu University, Zhenjiang Fourth People’s Hospital and Zhenjiang Women and Children’s Hospital, Zhenjiang, China; 2 Medical Center for Gynecology and Obstetrics, Fourth Affiliated Hospital of Jiangsu University, Zhenjiang Fourth People’s Hospital and Zhenjiang Women and Children’s Hospital, Zhenjiang, China; 3 Breast Surgery, Fourth Affiliated Hospital of Jiangsu University, Zhenjiang Fourth People’s Hospital and Zhenjiang Women and Children’s Hospital, Zhenjiang, China; 4 Reproductive Medicine Center, Fourth Affiliated Hospital of Jiangsu University, Zhenjiang Fourth People’s Hospital and Zhenjiang Women and Children’s Hospital, Zhenjiang, China

**Keywords:** biomarker, breast cancer, checkpoint proteins, chromosomal instability, lncRNA

## Abstract

**Objective:**

Chromosomal instability (CIN) drives genomic structural variants (such as amplifications and deletions) and promotes tumor progression. Long noncoding RNAs are known to participate in key biological processes and CIN regulation. Our previous study demonstrated that the highly expressed lncTRDMT1–5 serves as a novel prognostic biomarker in breast cancer; however, its underlying mechanisms require further investigation.

**Methods:**

LncTRDMT1–5 expression was knocked down in MDA-MB-231 cells, followed by chromosomal microarray analysis (CMA). Expression levels of lncTRDMT1–5 were examined in breast cancer cells and tissues via RT-PCR. Chromatin immunoprecipitation was used to detect H3K27 acetylation in the promoter region. RNA pull-down was performed to identified interactions with the MSRB3 protein. Cell proliferation was evaluated by EdU assay, DNA damage was assessed by γH2AX immunofluorescence, and cell cycle distribution was analyzed by flow cytometry. EMT-related protein expression and cell cycle regulators were examined by western blotting.

**Results:**

LncTRDMT1–5 was significantly upregulated in breast cancer cells and tissues, with H3K27 acetylation detected in its promoter. Downregulation of lncTRDMT1–5 induced CNVs across different chromosomes. Overexpression of lncTRDMT1–5 promoted cell proliferation, DNA damage, and EMT-related protein expression. RNA pulldown confirmed the relationship between lncTRDMT1–5 and MSRB3 protein. Mechanistically, lncTRDMT1–5 regulated cell cycle arrest by inhibiting p53 and inducing the expression of cell-division cycle protein 20 homologue (CDC20).

**Conclusion:**

Overexpression of lncTRDMT1–5 in breast cancer induces CIN and tumor progression by promoting EMT process and increasing MSRB3, p53 and CDC20 protein expression. Targeting this molecular pathway may offer novel therapeutic strategies and improve prognostic evaluations.

## 1 Introduction

Chromosomal instability (CIN) is widely recognized as a major cause of genomic structural variants, including sequence amplifications and deletions (1, 2). Widespread evidence has suggested that CIN is a common feature of cancer cells and plays an important role in tumor progression, metastasis, and chemoresistance (2, 3) via many potential factors, including the degree of CIN, the complex balance between the harmful and advantageous effects of CIN, and the diverse consequences of CIN (4). Copy number variations (CNVs) are a hallmark of CIN and lead to the gain or loss of chromosomal sections. For example, patients with carcinoma of unknown primary (CUP) exhibit different frequencies of CNV gains and losses, such as gains of 1q and 8q, and losses of 3p and 6q (5). Additionally, CIN in breast cancer (BC) usually predicts malignant progression (6); therefore, the identification of genetic mechanisms at the genomic level is useful.

Our previous study identified lncTRDMT1–5 as a novel prognostic biomarker in BC through bioinformatic analysis. We confirmed its significant upregulation in BC and suggested its potential involvement in key regulatory biological pathways (7). To explore the molecular pathway of lncTRDMT1–5 in BC, we found that the expression level of lncTRDMT1–5 was positively correlated with both vimentin (VIM) and MSRB3 levels in BC. Evidence suggests that VIM is associated with the epithelial-mesenchymal transition (EMT) process (8), whereas MSRB3 is related to genomic instability through ZEB1 interactions (9). Recent bioinformatics studies have demonstrated the potential role of lncTRDMT1-5 (LNCipedia: AL133415.1) in cancer progression, including its potential role as a ferroptosis-related lncRNA in bladder cancer immunotherapy (10) and its association with genomic instability in glioma (11). These observations remain largely predictive and lack experimental validation. Critically, the functional mechanisms of lncTRDMT1–5 in chemoresistance and DNA repair regulation in breast cancer remain unexplored. Thus, we hypothesize that lncTRDMT1–5 may be involved in CIN regulation.

Investigations to differential gene expression in cancer have extensively explored long noncoding RNAs (lncRNAs) that mediate CIN by targeting crucial checkpoint proteins or by acting through CIN-associated pathways (12). The extent of CIN may explain the exacerbation of cancer progression and the reduction in patient survival, which has motivated efforts to elucidate the CIN modulatory mechanism in tumors. For example, the lncRNA NORAD has been demonstrated to play a crucial role in the inhibition of cell proliferation and maintenance of CIN (13). Additionally, the downregulation of NORAD induces CIN in cell lines, and its level seems to increase following doxorubicin-induced DNA damage (14). Mechanistically, NORAD interacts with PUMILIO to influence a set of downstream processes including mitosis, DNA repair and DNA replication (15). In addition, previous reports have demonstrated that 8q24 possesses a large centromere protein A (CENP-A) domain, whereas it contains five unique lncRNAs (PCAT1, PCAT2, CCAT1, CCAT2 and PVT1). Previous evidence has shown that the loss of 8q24-derived lncRNA depletes the mislocalization of CENP-A and CENP-C in colon cancer cells (16). Moreover, the intrinsic pathology of BC progression and its targeted therapy have attracted substantial interest. Research has demonstrated diverse notions of the underlying mechanism of this disease, including mechanisms involving genomic instability and proteomic dysregulation. Furthermore, CIN has been shown to be positively correlated with BC prognosis (6).

In this study, we aimed to elucidate the functional role of lncTRDMT1–5 in BC progression and its potential involvement in CIN. We demonstrated that elevated lncTRDMT1–5 expression, driven by histone H3 lysine 27 acetylation (H3K27ac), promotes cell proliferation, EMT and exacerbated drug-induced DNA damage. Mechanistically, our findings suggest that lncTRDMT1-5 may influence cell cycle regulation and modulate downstream proteins such as AURKA, MSRB3 and p53. Collectively, these observations indicate that lncTRDMT1-5 may serve as a potential prognostic biomarker and that the associated pathway could offer novel therapeutic strategies for BC.

## 2 Material and methods

### 2.1 Patient tissue samples

This study contained 60 BC patients and 20 adjacent non-tumorous tissues who underwent surgical resection in the Fourth affiliated hospital of Jiangsu University (Zhenjiang, China) between January 2022 and December 2023. None of the patients received local or systemic therapy before surgery. The diagnosis was confirmed pathologically. Cases with unresolved pathological discrepancies were not enrolled. Tissues were rapidly frozen in liquid nitrogen and stored −80 °C until total RNA was extracted. The protocol was approved by the Ethics Committee of Fourth affiliated hospital of Jiangsu University (ethical number: 2021018). Written informed consent was obtained from all participants before further use.

### 2.2 Cell culture

The human breast cancer cells MCF-7, BT549, MDA-MB-231 and SKBR3 (Chinese Academy of Sciences, Shanghai, China. *Catalog No: TCHu-74, TCHu-93, TCHu-227, SCSP-5243, respectively*) were preserved in our laboratory. MCF-7 cells were cultured in Dulbecco’s modified Eagle’s medium (DMEM, HyClone) containing 10% fetal bovine serum (FBS; Thermo Fisher Scientific Inc.) and supplemented with 100 U/ml penicillin/streptomycin (Thermo Fisher Scientific, Inc.) and 10μg/ml insulin (Sigma-Aldrich). BT549, MDA-MB-231 and SKBR3 cells were cultured in Roswell Park Memorial Institute (RPMI) 1640 medium (HyClone) containing 10% FBS and supplemented with 100 U/ml penicillin/streptomycin. MCF-7/ADR cells (Shanghai Antique Biotechnology Company, China; Catalog No: BG006) are cultured from parental MCF-7 cells in RPMI-1640 medium with increasing concentrations of doxorubicin and supplemented with 100 U/ml penicillin/streptomycin. The cultures were incubated at 37 °C in a humidified 5% CO2 atmosphere.

### 2.3 Cell transfection

The full length of lncTRDMT1-5 (ENST0000456355) and MSRB3 were amplified and cloned the pcDNA3.1 vector (Ribo Biotech, Guangzhou, China), including empty vector (EV). The synthetic oligonucleotides used for silencing lncTRDMT1-5 (lncTRDMT1-5siRNA) and MSRB3 (MSRB3siRNA) were synthesized by Ribo™ lncRNA smarter silencer (Ribo Biotech, Guangzhou, China), including negative control (NC). Cells were transfected with the above vectors by using lipofectamine 3000 transfection kit (Invitrogen; Thermo Fisher Scientific, Inc.) according to the manufacturer’s protocol. A total of 4 × 10 (5) target cells were seeded into each well of a 6-well plate (in triplicate) and cocultured with vectors (2μg) or oligoribonucleotides (5μl) in the presence of 10μl lipofectamine and medium (total volume 250μl) upon reaching 70 to 80% confluence. The altered RNA/protein expression of target genes was measured after 24h or 48h of transfection. Each experiment was performed by triplicates.

### 2.4 Quantitative real-time polymerase chain reaction analysis

Total RNA from cells was isolated by RNAios plus (Takara, Tokyo, Japan). Total RNA was then reverse‐transcribed into complementary DNA (cDNA) through HiFiScript cDNA Synthesis Kit (CwBio, China). The reverse transcription (RT) protocol consisted of dNTP Mix (4μl), Primer Mix(2μl), RNA template(1μl), RT buffer(4μl), 1x DTT(2μl), 10mM HiFiScript (1μl), and RNase-Free Water(6μl) in a total volume of 20μl, then incubation at 42°C for 50 min followed by 85°C for 5 min. Then, quantitative real-time RT-PCR (qRT-PCR) analysis was performed in a total reaction volume of 20μl, comprising 10μl of SYBR Green Mix (2X), 2μl of cDNA template, 1μl of lncTRDMT1–5 forward primer (10μM), 1μl of lncTRDMT1–5 reverse primer (10μM), and 6μl of RNA-free water. The qRT-PCR cycle settings included an initial denaturation step of 5 min at 95°C; 40 cycles of 15 s at 95°C and 30 s at 60°C, and a final extension step of 60 s at 60°C; reactions were performed with a Bio-Rad CFX96 instrument (ABI Corporation, CA, United States). The primers were purchased from Ribo Biotechnology (Guangzhou, China). The primer sequences for qRT-PCR in our study were as follows: ENST00000456355 F: 5-GGGCTGCCTTACCCTCATT-3, R: 5-GCCATACACTTTTACATCCTCCAT-3; Experiments were repeated three times and the relative RNA expression was calculated using the 2^−ΔΔCt^ method, with GAPDH as the internal reference gene for normalization.

### 2.5 Edu assay

MCF-7 and MDA-MB-231 cells were seeded into 24‐well plates at a cell quantity of 4 × 10 (3) per well, incubated at 37°C for 24h, transfected with siRNA and cultured for 48h. Then 200μl EdU labeling medium was added to cells 48h after transfection using Cell Light EdU Apollo488 *In Vitro* kit (Guangzhou RiboBio Co., Ltd.), and they were incubated for 2 h at 37 °C under 5% CO_2_. Next, the cultured cells were treated with 4% paraformaldehyde for 30 min and then 100μl 0.5% Triton X-100 for 10 min at room temperature. Then the samples were stained with Apollo staining solution and subsequently incubated with 100μl Hoechst 33342 (5μg/ml). Five fields of view were randomly selected in each well for counting the percentage of EdU-positive cells. The percentage of EdU-positive cells was measured under fluorescent microscopy.

### 2.6 H2AX assay and cell cycle analysis

MCF-7 and MDA-MB-231 cells seeded on 24-well plates and washed by PBS according to the different experimental conditions (treated with doxorubicin (0.25 Mm) or lncTRDMT1–5 plasmid), and fixed with 4% formaldehyde for 10 min. Samples were then washed three times with phosphate-buffered saline (PBS) and blocked for 20 min in PBS containing 1% BSA, incubated overnight at 4°C with H2AX antibody (Abnova, Taiwan), washed with PBS, and incubated for 2 h at RT with Cy3-antibody (BBI, Shanghai). Finally washed again and incubated for 5min at RT with DAPI. Images were acquired using a confocal microscope. For cell cycle analysis, MCF-7 and MDA-MB-231 cells were transfected with lncTRDMT1–5 plasmid and siRNA, as well as EV and NC respectively. Cells were incubated for 72 h, and then collected and resuspended in cold ethanol (70%) overnight at 4 °C. The cells were pelleted and then incubated with working solution (10 μl/per propidium iodide and RNase A) for 30 min at 37 °C in the dark (Servicebio, Wuhan, China). Experiments were repeated three times and cell cycle was evaluated using a flow cytometer (FACS Calibur, BD Biosciences, USA).

### 2.7 Fluorescence *in situ* hybridization analysis

The RNA FISH probe mix for lncTRDMT1–5 was designed and synthesized by RiboBio for FISH analysis in MDA-MB-231 cells. Cells were fixed with 4% formaldehyde and washed with PBS, then 0.5% Triton X-100 was added for 5 min at 4°C. The fixed cells were incubated with a solution containing pre-hybridization buffer at 37°C for 30min, then incubated with 20 μM of the FISH probe mix (RiboBio, China) in 100 μl hybridization buffer overnight at 37°C. After hybridization, the coverslips were washed with hybridization buffer for different concentration and incubated with DAPI for 10 min. Images were visualized with confocal microscope (LSM710, Carl Zeiss).

### 2.8 Western blot analysis

Transfected MCF-7 and MDA-MB-231 cell lysates from RIPA buffer. A total of 100μg of protein from each sample was mixed with protein loading buffer (5X), and separated on an 8-10% SDS-polyacrylamide gel, then transferred to 0.45μm PVDF membranes (Millipore, USA). Samples on the membranes were sealed with 5% non-fat dry milk for 1h at room temperature, and then incubated with primary antibodies, including E-cadherin (Proteintech, 20874-1-AP, 1:20000), VIM (Proteintech, 60330-A-Ig, 1:20000), MSRB3 (Nature Biosciences, A13326), GAPDH (Santa Cruz Biotechnology, sc-47724, 1:1000), AURKA (Proteintech, 66757-1-Ig, 1:1000), CDC20 (Proteintech, 10252-1-AP, 1:2000), and p53 (Proteintech, 10442-1-AP, 1:5000) at 4°C overnight. The membranes were washed with agitation in 0.1% Tween 20 in TBS and incubated with HRP-conjugated secondary antibodies (BBI, D110087, and D110058, 1:5000 or 1:10000) for 2h at room temperature. Finally, membranes were incubated with enhanced chemiluminescence (ECL) reagent (Beyotime, Jiangsu). The result was visualized by the ChemiDOC Image system (BioRad, USA). Densitometry analysis was performed using ImageJ software. Data were normalized using the GAPDH protein levels. Fold changes in the intensity of the protein signals were reported as the mean of the results from three experiments.

### 2.9 Chromatin immunoprecipitation assay

ChIP assays were performed with MCF-7 and MCF-7/ADR cells using EZ-Magna ChIP kit (Millipore) according to the manufacturer’s protocol. In brief, MCF-7/ADR cells with 1% of formaldehyde were incubated for 10 minutes to form DNA‐protein crosslinks, and then cross‐linked chromatin DNAs were sonicated into 200 to 300 bp‐sized segments and immunoprecipitated with H3K27 antibody (Abcam) and IgG antibody was used to precipitate the chromatin the lysate. ChIP samples were analyzed via qPCR. C646, an acetylation inhibitor, was purchased from Sigma Chemical and dissolved in enclosed sterile water.

### 2.10 RNA pull down assay

The protein extracts from MDA-MB-231 cells were treated with biotinylated RNA (lncTRDMT1–5 biotin probe) and beads for recovering, with no-biotin probe as control. WB was operated to detect the enrichment of methionine sulfoxide reductase B3 (MSRB3) antibody (Nature Bioscience, A61066) and Aurora Kinase B (AURKB) antibody (Nature Bioscience, A13326) in RNA-protein complex.

### 2.11 Chromosomal microarray analysis

Microarray analysis was performed on DNA isolated from the MDA-MB-231/NC and MDA-MB-231/lncTRDMT1-5siRNA. 750K (Affymetrix, Inc., Santa Clara, CA) was applied in each group to better elucidate the effects of lncTRDMT1–5 downregulation on chromosomal deletion/amplification. The array contains more than 2.4 million markers for copy number evaluation and approximately 750,000 single nucleotide polymorphisms (SNPs). The results were analyzed by Affymetrix Chromosome Analysis Suite (ChAS) software. Map position was based on the UCSC Genome Browser, GRCh38 (NCBI Build 38 reference sequence).

### 2.12 Statistical analyses

All data are presented as mean ± SD from at least three independent experiments. All statistical analyses were conducted using GraphPad Prism 8.0. Normality of data distribution was assessed using Shapiro-Wilk test. For comparisons between two groups, the unpaired Student’s t-test was used for parametric data, while the Mann-Whitney U test was applied for non-parametric data. For comparisons involving more than two groups, one-way ANOVA or two-way ANOVA was performed, followed by Sidak’s *post hoc* test for multiple comparisons. A P value < 0.05 was considered statistically significant.

## 3 Results

### 3.1 LncTRDMT1–5 is involved in BC tumorigenesis and transcriptionally activated by H3K27 acetylation

**Figure 1:**
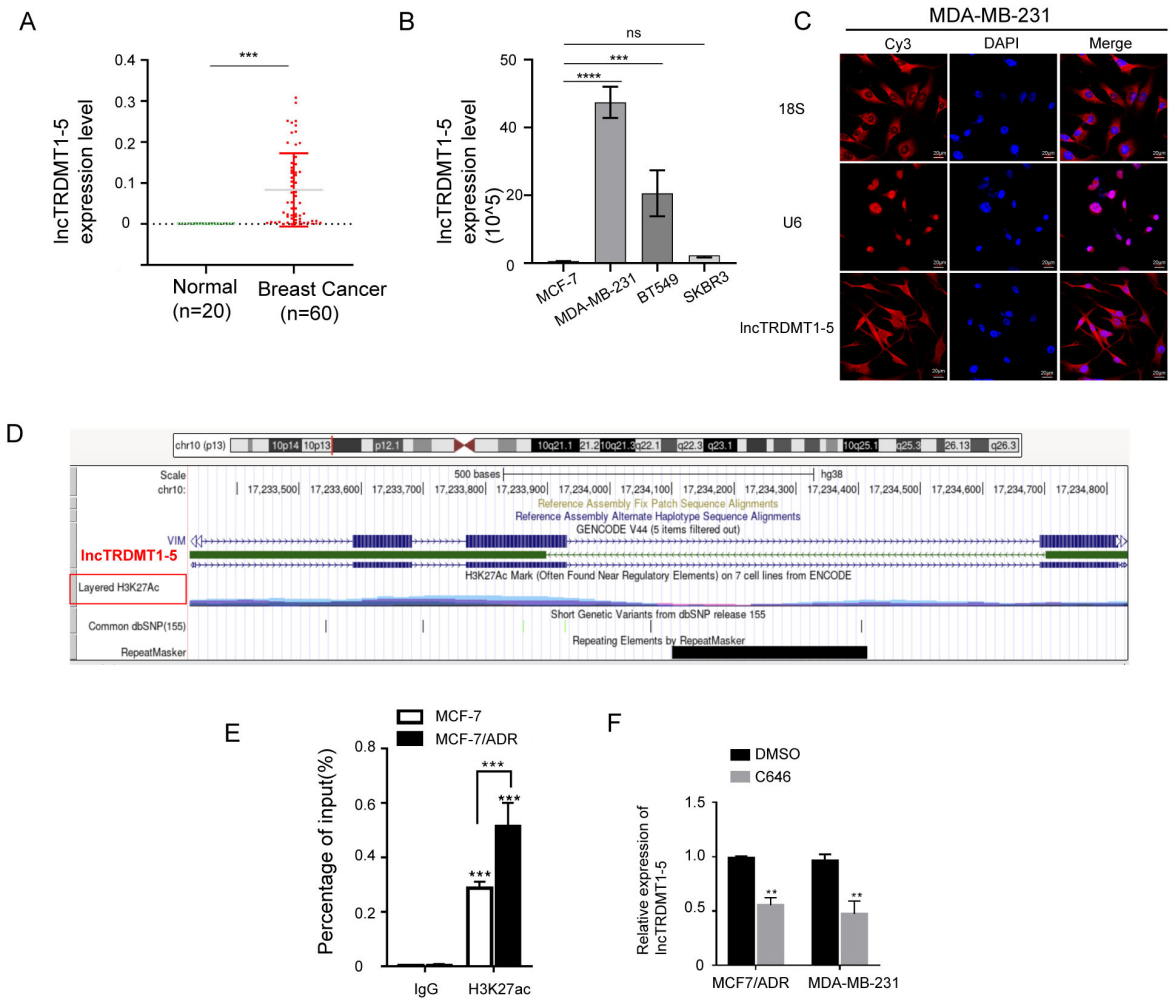
LncTRDMT1–5 is upregulated in TNBC cells and activated by H3K27ac. **(A)** RT-PCR was performed to measure the differential expression of lncTRDMT1–5 in BC tissues (n=60) compared to normal tissues (n=20). Statistical significance was determined using the Mann-Whitney U test. ***P < 0.001. **(B)** LncTRDMT1–5 expression varied in different subtypes of BC cells. MDA-MB-231 displayed a highest level compared to the other cell lines, including MCF-7, BT549, and SKBR3. One-way ANOVA followed by Tukey’s *post hoc* test was used for statistical analysis. ****P<0.0001. **(C)** FISH analysis of the subcellular location of lncTRDMT1–5 with specific probe in MDA-MB-231 cells. **(D)** The genome bioinformatics analysis in UCSC showed that the promoter of lncTRDMT1–5 had a high enrichment of H3K27ac. **(E)** The H3K27ac enrichment at lncTRDMT1–5 promoter was measured by ChIP assay in both MCF-7/ADR cells and MCF-7 cells. Two-way ANOVA followed by Sidak’s *post hoc* test was used for statistical analysis. *P<0.05 compared to parental cells. **(F)** lncTRDMT1–5 expression was measured by qPCR in BC cells treated with C646 or DMSO for 48 h. Statistical significance was determined using Welch’s t-test. **P < 0.01 compared to DMSO treatment group.

To further analyze the clinical characteristics of lncTRDMT1–5 in BC, we performed PCR analysis of lncTRDMT1–5 in patient samples that included 60 BC tissue samples. We demonstrated significantly higher levels of lncTRDMT1–5 in BC tissues (*p* < 0.001) than in normal tissues (**Figure 1A**). This significantly upregulated lncTRDMT1–5 expression confirms in our previous study findings. In addition, detailed clinical correlation analysis revealed that a high level of lncTRDMT1–5 was associated with lymph node metastasis and distant metastasis (7) (data not shown). LncTRDMT1–5 expression was higher in triple-negative breast cancer (TNBC) patient samples than in luminal (p<0.001) and human epidermal growth factor receptor 2 (HER2) subtype ones (p<0.001). In agreement, MDA-MB-231 cells presented the highest expression of lncTRDMT1-5, whereas the MCF-7 cells exhibited the lowest expression (**Figure 1B**). This result is consistent with our previous study, which focused on lncTRDMT1–5 expression among different subtypes of BC tissues (7). To explore potential mechanism, its subcellular localization was analyzed with FISH assays that lncTRDMT1–5 was distributed in both the cytoplasm and nucleus (**Figure 1C**), suggesting that it may participate in the processes of transcription and posttranscriptional regulation. To analyze lncTRDMT1–5 upstream regulation, we explored its promoter region using the University of California Santa Cruz (UCSC) Genome Browser (http://genome.ucsc.edu/), and identified strong enrichment of H3K27ac mark (**Figure 1D**). We subsequently conducted ChIP assays to verify the occurrence of histone acetylation. As presented in **Figure 1E**, both cell lines exhibited H3K27ac enrichment in the promoter region of lncTRDMT1-5(p<0.001). Moreover, the enrichment of H3K27ac was markedly greater in MCF-7/ADR cells than in parental cells(p<0.001). In addition, treatment with C646 (a histone acetyltransferase inhibitor) significantly decreased the expression level of lncTRDMT1-5 (**Figure 1F**, *p* = 0.008). Taken together, lncTRDMT1–5 augmented level in BC cells may be due to increased histone acetylation in its promoter region.

### 3.2 Effects of lncTRDMT1–5 on the proliferation, cell cycle and EMT in BC cells

**Figure 2:**
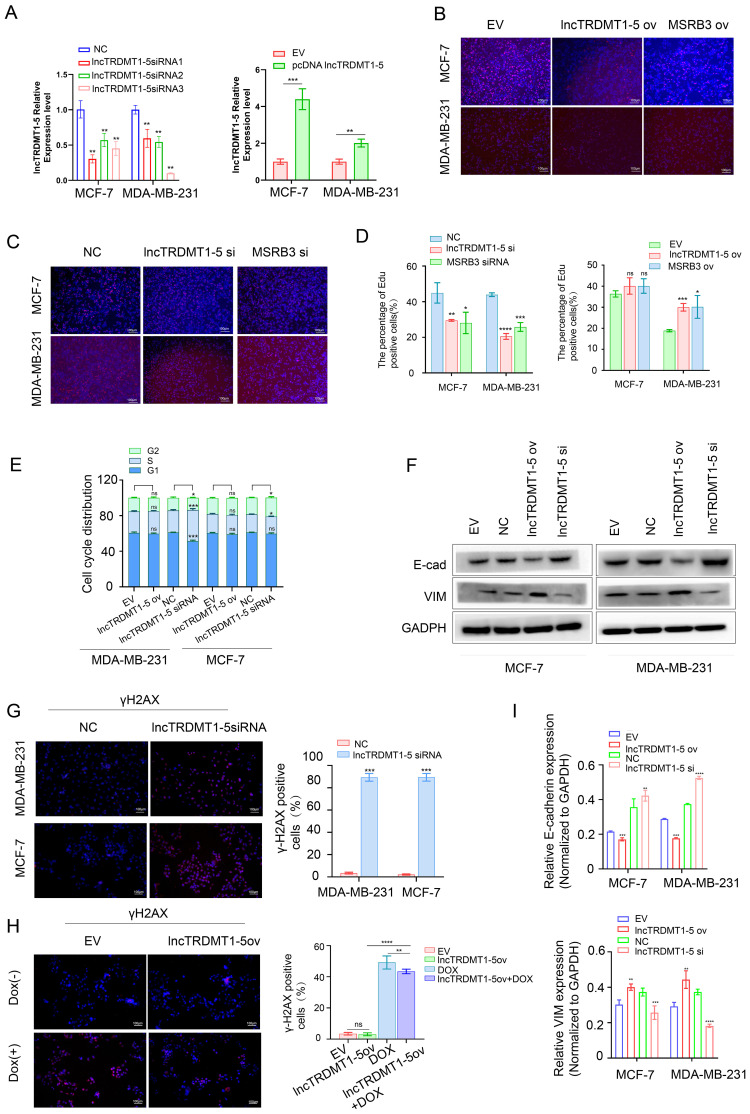
Silencing lncTRDMT1–5 inhibits cell proliferation and cell cycle. **(A)** The expression levels of lncTRDMT1–5 in both MDA-MB-231 and MCF-7 cell lines were measured by RT-PCR after transfection with either pcDNA vector or specific siRNAs. **(B, C)** Edu-positive cells were detected after transfection using Edu staining. **(D)** The percentage of Edu-positive cells was quantified through Edu staining in MCF-7 and MDA-MB-231 cells transfected with lncTRDMT1–5 plasmids and siRNAs. Data are represented as the mean ± SEM (n = 3 independent experiments). Statistical significance was determined using Welch’s t-test. **(E)** Flow cytometric analysis was performed to evaluate cell cycle in MCF-7 and MDA-MB-231 cells transfected with either lncTRDMT1–5 plasmids or siRNAs, with empty vector (EV) and scrambled siRNA (NC) serving as corresponding controls. **(F, I)** Western blot analysis was performed to measure the expression of epithelial-mesenchymal transition related proteins, including E-cadherin and VIM. GAPDH was used as a control. The fold of VIM and E-cadherin was normalized to EV or NC, respectively **(I)**. Three independent experiments were performed, and the P values were calculated by Student’s t test. **(G)** Detection of γH2AX foci 48h after transfection of lncTRDMT1-5siRNA in MDA-MB-231 and MCF-7 cells. Statistical significance was determined using Welch’s t-test. Quantification of 3 fields with 100 cells total. Scale bars: 100μm. **(H)** Doxorubicin-induced DNA damage in control and MCF-7 cells with forced expression of lncTRDMT1–5 as measured by immunofluorescence staining. Representative picture of γH2AX-positive cells, Scale bars, 100μm. Statistical significance was determined using Two-way ANOVA; *p < 0.05, **p < 0.01 ***P < 0.001. ****P < 0.0001.

To identify the function of lncTRDMT1–5 in BC progression, we performed a loss/gain-of-function assay with MCF-7 and MDA-MB-231 cells (**Figure 2A**). EdU analysis demonstrated the impact of lncTRDMT1–5 depletion on cell proliferation (**Figures 2B, C**). Moreover, a significant decrease in viability was observed after knockdown of lncTRDMT1–5 in MCF-7 (*p* = 0.009) and MDA-MB-231 cells (*p* < 0.001), whereas lncTRDMT1–5 overexpression significantly increased the number of Edu-positive cells in MDA-MB-231 cells (*p* < 0.001) (**Figure 2D**). Afterwards, we used flow cytometry to analyze the cell cycle characteristics of BC cells to explore the underlying mechanism of action of lncTRDMT1-5. The results revealed a significant decrease in the percentage of cells in the G1 phase and an increase in the percentage of cells in the S phase after lncTRDMT1–5 knockdown in MDA-MB-231 cells. However, in MCF-7 cells, downregulation of lncTRMDT1–5 led to a decrease in the number of cells in the S phase, but an increase in the number of cells in the G2 phase (**Figure 2E**). However, there was no significant effect in MCF-7 and MDA-MB-231 cell cycle by lncTRDMT1–5 upregulation (**Figure 2E**). These data suggested that the knockdown of lncTRDMT1–5 induced cell cycle arrest in the G1 or S phase in different cell lines. Our previous study demonstrated a positive correlation between lncTRDMT1–5 and VIM expression levels. We hypothesized that lncTRDMT1–5 functionally regulates the EMT process. We subsequently assessed whether lncTRDMT1–5 could regulate EMT biomarkers in MCF-7 and MDA-MB-231 cells. As shown in **Figures 2F, I**, lncTRDMT1–5 overexpression significantly increased the protein levels of VIM, and decreased the protein level of E-cadherin compared with those in control cells. As expected, VIM expression was downregulated, whereas the E-cadherin level was increased in lncTRDMT1-5-silenced cells compared with control cells (**Figure 2I**). Moreover, we observed that the downregulation of lncTRDMT1–5 induced DNA damage, as evidenced by a significant increase in γh2AX-positive cells compared with parental cells (**Figure 2G**, *p* < 0.001)). When considering that doxorubicin (Dox) is associated with DNA damage, we determined the degree of DNA damage in MCF-7 cells overexpressing lncTRDMT1–5 with or without Dox treatment. In the EV group, Dox induced significant DNA damage. However, lncTRDMT1-5-overexpressing cells did not exhibit significant DNA damage in the absence of Dox compared with the EV-Dox(-) group (*p* = 0.990). Notably, in lncTRDMT1-5-overexpressing cells treated with Dox, the extent of DNA damage decreased toward baseline (*p* < 0.001). These results indicated that DNA damage was reversed as a result of lncTRDMT1–5 overexpression (**Figure 2H**).

### 3.3 LncTRDMT1–5 regulates the expression of MSRB3 involved in cell cycle and DNA damage

**Figure 3:**
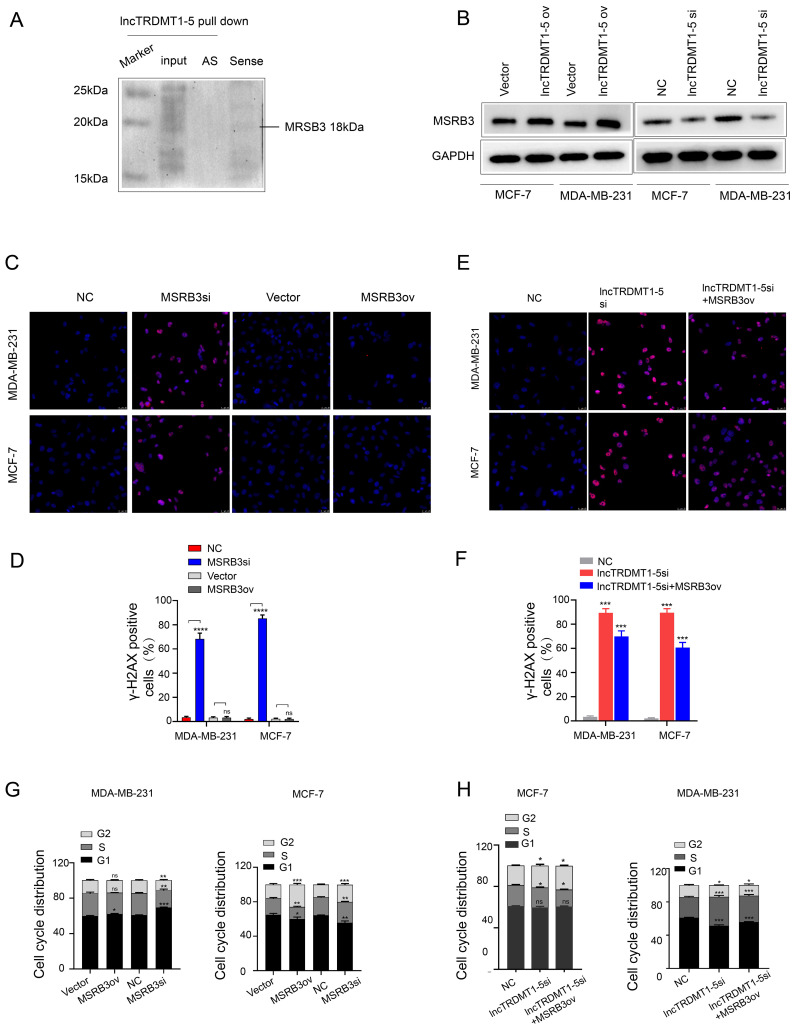
LncTRDMT1–5 regulates the expression of MSRB3 involved in cell cycle and DNA damage. **(A)** RNA pulldown analysis showed the interaction between lncTRDMT1–5 and MSRB3 protein. Full-length blots are presented. **(B)** WB analysis showed that MSRB3 protein level in MDA-MB-231 and MCF-7 cells transfected with lncTRDMT1–5 plasmid or siRNA, or control vectors, respectively. **(C, D)** Detection of γH2AX-positive cells by immunofluorescence assay in MDA-MB-231 cells transfected with MSRB3 plasmid or siRNA, or control vectors. **(E, F)** Detection of γH2AX-positive cells in lncTRDMT1-5-knockdown MDA-MB-231 and MCF-7 cells transfected with MSRB3 plasmid. **(G)** FACS analysis was subjected to assess cell-cycle distribution of MCF-7 and MDA-MB-231 cells with MSRB3 overexpression or knockdown. **(H)** Cell cycle distribution in lncTRDMT1-5-knockdown MDA-MB-231 and MCF-7 cells transfected with MSRB3 plasmid. Three independent experiments were performed for quantification (mean ± SD). Unpaired two-tailed Student’s t-test was used for statistical analysis. *P < 0.05, **P < 0.01 ***P < 0.001. ****P < 0.0001.

Given that MSRB3 participates in cell proliferation (17), cell cycle regulation and apoptosis (18), as well as the fact that MSRB3 mRNA levels are positively correlated with lncTRDMT1–5 based on our previous findings (7), we explored the potential effects of lncTRDMT1–5 on MSRB3 expression and investigated whether dysregulation of lncTRDMT1–5 affects CIN traits in BC cells. In our previous study, we constructed a correlation network comprising lncTRDMT1–5 and mRNAs based on correlation scores. Through this analysis, MSRB3 was identified as a candidate target due to its critical roles in cell cycle regulation and apoptosis, prompting us to further investigate its potential interaction with lncTRDMT1-5 (7). WB analysis was performed to verify the expression of downstream proteins. A pull-down assay combined with WB analysis was used to examine the interaction between lncTRDMT1–5 and the downstream protein MSRB3. We observed that MSRB3 was enriched in the lncTRDMT1–5 pull-down fraction, suggesting that MSRB3 associates with lncTRDMT1-5 (**Figure 3A**). In addition, the AURKA and MSRB3 protein levels were decreased after the knockdown of lncTRDMT1–5 in MDA-MB-231 and MCF-7 cells, whereas overexpression of lncTRDMT1–5 increased MSRB3 protein levels (**Figure 3B**). These findings suggest that MSRB3 contributes to the biological effects of lncTRDMT1–5 during mitosis. lncTRDMT1–5 mainly performs its biological function mainly via MSRB3 during mitosis. Afterwards, cell cycle analysis revealed that compared with that of untreated cells, the proportion of MCF-7 cells in the S/G2 phase was increased after MSRB3 overexpression; additionally, the proportion of MDA-MB-231 cells in the G1/S phase was increased. Specifically, MSRB3 knockdown in MDA-MB-231 cells increased the proportion of cells in the G1 phase and decreased the proportion of cells in the S phase, whereas decreased expression of MSRB3 in MCF-7 cells decreased the proportion of cells in the G1 phase but increased the proportion of cells in the S/G2 phase (**Figure 3G**). DNA damage analysis revealed that MSRB3 knockdown led to a significantly greater number of positive MDA-MB-231 and MCF-7 cells (*p* < 0.001), whereas the overexpression of lncTRDMT1–5 did not result in obvious DNA damage (**Figures 3C, D**). Next, we determined whether lncTRDMT1–5 regulates CIN by modulating MSRB3. For this rescue experiment, we overexpressed MSRB3 in lncTRDMT1-5-knockdown MDA-MB-231 cells and MCF-7 cells. The results demonstrated that MSRB3 overexpression abrogated the suppressive effects of lncTRDMT1–5 knockdown on cell cycle arrest (**Figure 3H**) and DNA damage in both MDA-MB-231 and MCF-7 cells (**Figures 3E, F**, *p* < 0.001)). These findings suggest that lncTRDMT1–5 regulates MSRB3 expression, which in turn modulates cell cycle progression and DNA damage responses, indicating a mediating role for MSRB3.

### 3.4 LncTRDMT1–5 knockdown is associated with altered CNV profiles and induces p53 level in BC cells

**Figure 4:**
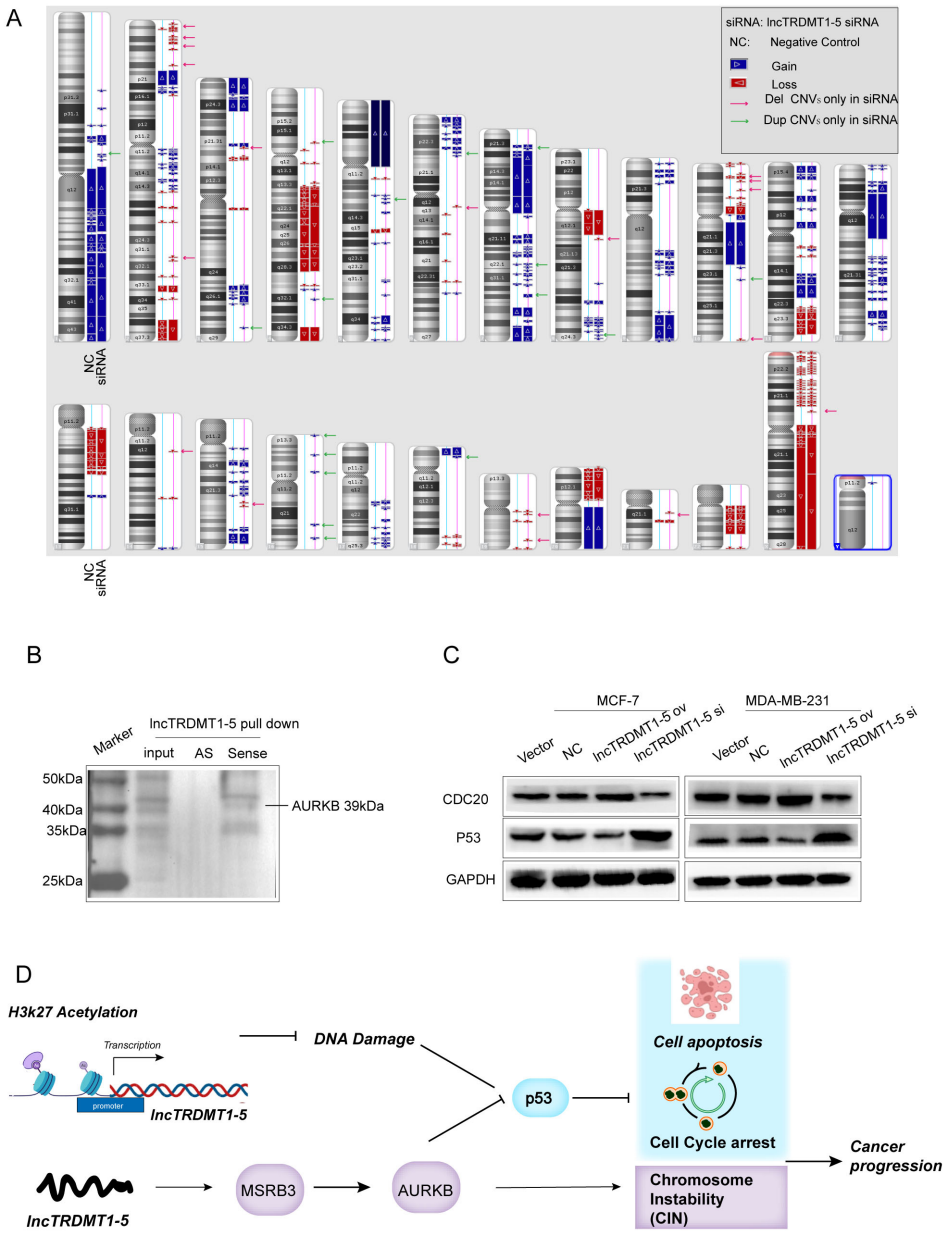
Silencing lncTRDMT1–5 induced p53 level and overcame CIN. **(A)** chromosomal aberrations across the whole genome by CMA analysis. Affymetrix CytoScan 750K Array analysis, using genomic DNA from MDA-MB-231 cells and lncTRDMT1–5 knockdown cells. Blue box represents gains; red box represents deletions. Red arrows indicate deletions of CNVs and green arrows show duplications of CNVs only existing in siRNA groups. Chromosomes are arranged in numerical order (1-22, X), with chromosome numbers indicated at the lower left of each ideogram. **(B)** RNA pulldown analysis showed the interaction between lncTRDMT1–5 and AURKB protein. **(C)** WB analysis of AURKA, CDC20 and p53 expression in MDA-MB-231 cells and MCF-7 cells with downregulated lncTRDMT1-5. For cells with shRNA compared with control. **(D)** Mechanism illustration.**Supplementary Table 1**. Segments details (loss and gain) exist only in negative-control cells.

CIN is a common feature of BC and is associated with patient prognosis. To further explore the relationship between lncTRDMT1–5 and CIN, we performed CMA analysis using lncTRDMT1–5 siRNA in MDA-MB-231 cells. The genomic location of the probe is shown on the vertical axis of the corresponding **Figure 4A**. The CMA results revealed a discrepancy in the number of loss-/gain-CNVs between lncTRDMT1-5-knockdown cells and control cells (**Figure 4A**, **Supplementary Table 1**). Notably, several high-risk chromosomal variations, including deletions in chromosome 11q23.3 (CCDC84, H2AX), and gains in chromosomes 2q11.2q12.1 (CNOT11), 2q13 (BUB1), 6p22.1 (H2BC13/14/15/17), 7q22.1 (PSMC2), 12q24.31 (CLIP1), 16p13.11 (NDE1), 17q23.1 (BRIP1), and 11q13.1 (KAT5), were observed in the control cells but not in the lncTRDMT1–5 siRNA cells (**Supplementary Table 1**). These findings suggested that lncTRMDT1–5 may be involved in the cell cycle checkpoint and G2/M checkpoint pathways. To examine the interaction between lncTRDMT1–5 and AURKB, we performed an RNA pull-down assay followed by western blotting with an anti-AURKB antibody. As shown in **Figure 4B**, AURKB was enriched in the lncTRDMT1–5 pull-down fraction, suggesting that AURKB associates with lncTRDMT1-5 (**Figure 4B**). In addition, various cell cycle checkpoint proteins, including AURKA, cell-division cycle protein 20 (CDC20), and p53, play crucial roles in the cell cycle and CIN regulation. AURKA also acts as a TP53-regulated transcription factor for cell cycle genes. To confirm the levels of these CIN-related proteins, a WB assay was used to verify whether lncTRDMT1–5 affects the levels of these proteins. After the knockdown of lncTRDMT1-5, AURKA and CDC20 levels were decreased in MCF-7 and MDA-MB-231 cells; however, the p53 level was increased. Moreover, opposite effects regarding the expression levels of AURKA, CDC20 and p53 were observed in lncTRDMT1-5-ov cells (**Figures 3B, C**). These results suggest that lncTRDMT1–5 influences CIN-related phenotypes and protein expressions, potentially involving AURKB and p53-associated pathways (**Figure 4D**). However, the detailed mechanisms remain to be elucidated.

## 4 Discussion

CIN drives tumor progression partially through genomic CNVs that are caused via alterations in the doses and expressions of genes located on imbalanced chromosomes (6). This instability mainly arises due to errors in DNA replication and chromosome segregation during cell division, thereby resulting in cells with numeric and/or structural aberrations (19). Notably, genetic variations in spindle assembly checkpoint (SAC) components or spindle attachment proteins can induce segregation defects, thus contributing to variable tumorigenesis (20). Although lncRNAs have emerged as critical regulators of CIN and useful therapeutic targets (12, 15, 21, 22), none of these lncRNAs have been approved for clinical applications. Herein, we identified lncTRDMT1–5 as a novel epigenetic mediator of CIN in BC. Through integrative bioinformatics analysis and ChIP assays, we demonstrated that the upregulation of this lncRNA was partially driven by H3K27ac activation. Other lncRNAs (e.g., MALAT1, GAS5, and H19) modulate tumor progression by promoting or inhibiting the epithelial-mesenchymal transition (EMT) (23–25). Our findings first elucidated that lncTRDMT1–5 not only promotes the EMT process, but also induces CIN by impairing the DNA damage response and modulating cell cycle arrest. Specifically, it promoted CDC20 expression and suppressed p53 expression (**Figure 4D**). Based on previous findings indicating that high lncTRDMT1–5 levels are associated with BC progression and poor prognosis (7), these findings not only expand the functional repertoire of lncRNAs in CIN, but also position lncTRDMT1–5 as a promising therapeutic target to reduce BC aggressiveness and improve patient outcomes.

The functional impact of CIN in cancer is context dependent, exhibiting either oncogenic or tumor-suppressive effects based on the specific carcinogenesis model and CIN drivers that are involved (19, 26). Several lncRNAs function as critical modulators of CIN, and their dysregulation often correlates with tumor progression (15, 21, 22, 27). For instance, the overexpression of CCAT2 promotes colorectal cancer aggressiveness by inducing structural and numerical chromosomal alterations; moreover, this phenotype is linked to the effects of CCAT2 in stabilizing BOP1 ribosomal biogenesis factor (BOP1) and subsequently activating aurora kinase B(AURKB), which is a key regulator of mitotic fidelity (21). In contrast, NORAD and GUARDIN are lncRNAs that maintain genomic stability; specifically, NORAD acts as a p53-dependent DNA damage responder and suppresses pumilio protein 1/2 (PUM1/2)-mediated mitotic dysfunction (including tetraploidization and mitotic defects) (13, 15), whereas GUARDIN safeguards genomic integrity by sustaining the expression of telomeric repeat-binding factor 2 (TRF2) (22). These paradoxical roles underscore the dual features of lncRNA-mediated CIN regulation in cancer. Despite these advances, only a few lncRNAs have been regarded as participating in CIN, and the knowledge regarding the mechanisms of CIN-associated lncRNAs remains limited. Our study supplements these theoretical strategies by identifying lncTRDMT1–5 as an oncogenic CIN driver in BC. Unlike CCAT2 or GUARDIN, lncTRDMT1–5 disrupts chromosomal segregation after the induction of DNA damage, thus resulting in cell cycle arrest, which suggests a distinct pathway for CIN induction.

DNA damage and CIN are closely intertwined. Replication stress-induced DNA double-strand breaks (DSBs) can drive the onset of CIN; conversely, CIN can also arise as a downstream consequence of DNA damage, as reported for NORAD (28) and LIMp27 (29), are upregulated upon DNA damage and contribute to genomic instability by disrupting cell proliferation and division. H19 promotes DNA damage repair and is correlated with BC progression and prognosis (30). In addition, errors in the DNA damage response may lead to chromosomal aberrations, thus resulting in the amplification and deletion of oncogenes or suppressor genes (19). However, the function of lncTRDMT1–5 in DNA damage repair has not been previously reported. To address this issue, the intensity of H2AX immunostaining was examined. Our data showed that lncTRDMT1–5 expression was significantly increased in MDA-MB-231 cells with doxorubicin-induced DNA damage. In general, cell cycle checkpoints elicit pauses in the cell cycle to maintain genome stability in response to DNA damage (29). Our data revealed that the number of cells in the G0/G1 phase decreased, along with the numbers of cells increasing in the G2/M phase after lncTRDMT1–5 knockdown. In our study, we demonstrated that cell cycle arrest activated by lncTRDMT1–5 can also result in decreased p53 levels, which can also be influenced by DNA damage. As shown in **Figure 3G**, we found that cell-cycle responses to MSRB3 knockdown in MDA-MB-231 and MCF-7 cells are fundamentally different, suggesting that MSRB3 may regulate cell cycle progression through distinct pathway in a cell-context-independent manner. These different responses are likely attributable to the intrinsic differences between the two cell lines, including p53 status (mutant in MDA-MB-231 vs. wild-type in MCF-7) (31, 32) hormone receptor profiles (triple-negative MDA-MB-231 vs. ER-positive MCF-7) (33), and other regulatory networks (34, 35).

Chromosome missegregation and DNA damage may trigger the activation of p53, which is a key tumor suppressor and “safeguard” that determines the cellular fate in response to CIN (36). The activation of p53 and its pathway leads to aneuploidy (4) and induces cell cycle arrest, apoptosis (20), or DNA damage repair (37), thereby preventing uncontrolled proliferation and maintaining genomic integrity (36, 38, 39). In response to DNA damage, p53 activation is achieved by disrupting the interactions between p53 and its functional genes, thereby leading to p53 stabilization and targeted gene regulation (37). Previous studies have demonstrated that high levels of DNA damage result in a sustained p53 response and apoptosis, whereas low levels of DNA damage lead to pulsed activation and milder effects, such as cell cycle arrest (40).

However, the deletion or mutation of p53 results in chromosomal instability, and the relationship of p53 with various cancers requires further validation (4). Previous studies have demonstrated that high expression levels of lncRNAs, such as cohesion regulator noncoding RNA (CONCR) and NORAD, are required for cell cycle regulation (28) and DNA replication in many types of cancers (41). Specifically, CONCR is indirectly decreased by p53 and participates in DNA replication. The depletion of CONCR can lead to the loss of sister chromatid cohesion, decreased cell proliferation and increased cell apoptosis (41). In addition, in gastric and colorectal cancer, mutant p53 (TP53) tumors present more unstable and complex karyotypes than p53-proficient tumors (42, 43). The ablation of TP53 *in vivo* is associated with cancer progression exhibiting increased CIN and overall aneuploidy (20). These findings suggest that loss of p53 function is correlated with supernumerary centrosomes in human cancers, thereby leading to CIN due to lagging anaphase chromosomes (44). Cells with p53 knockdown display multipolar spindles and are predisposed to whole-genome doubling (WGD) due to the survival of cells that undergo cytokinesis failure (45). Thus, in most cancers exhibiting early WGD, near-triploidy and extensive copy number alterations (CNAs) occur. Our results demonstrated that the overexpression of lncTRDMT1–5 caused negative regulation of p53 expression, thereby influencing cell cycle arrest and leading to CIN. Overall, p53 acts as a central regulator in balancing cell survival and death under genomic stress, whereas lncRNAs modulate these pathways in ways that can either suppress or promote cancer (depending on their functional roles). This intricate network underscores the importance of p53 and lncRNAs in cancer biology and their potential as therapeutic targets.

Chromosome segregation errors primarily arise due to defects in the mitotic machinery, including centrosome amplification, faulty microtubule kinetochore attachments, SAC dysregulation, or impaired sister chromatid cohesion (2, 3). The SAC functions as a crucial surveillance system that ensures proper chromosome alignment by monitoring kinetochore attachment and delaying mitotic progression when errors are detected (46). When SAC activity is compromised, chromosomes are misinterpreted and fail to inhibit the anaphase-promoting complex bound to its activator subunit Cdc20 (APC/CCdc20), thereby leading to premature chromosome segregation and aneuploidy (47). Our findings revealed that lncTRDMT1-5, which is activated by H3K27ac, acts as a potential oncogene by disrupting key chromosomal segregation checkpoints, including the SACs and DNA damage response pathways. Specifically, lncTRDMT1–5 overexpression upregulated the expression of critical mitotic regulators, including AURKA, AURKB, and CDC20. Notably, AURKA overexpression *in vivo* promotes aneuploidy and chromosome missegregation (48), whereas CDC20 inhibition exhibits tumor-suppressive effects (49). These findings highlight the therapeutic potential of the targeting of these pathways, as evidenced by the fact that several AURKA/B/C inhibitors are currently in phase I-III clinical trials for CIN-based anticancer therapy (19). Our findings underscore the importance of mitotic regulation in maintaining genomic stability and suggest that lncTRDMT1-5-mediated disruption of these processes contributes to tumorigenesis.

Recent literatures have revealed that the elevation of CIN levels beyond a tolerable threshold can selectively induce cell death, thereby indicating that making CIN modulation is a promising therapeutic strategy. Current research has focused on two main therapeutic strategies: (1) the evaluation of CIN levels in cancer cells to identify vulnerabilities and (2) the targeting of survival mechanisms that allow cancer cells to tolerate chromosome missegregation. This approach has been validated through multiple studies demonstrating that the targeting of CIN-related pathways can overcome drug resistance and suppress tumor growth. For instance, CONCR knockdown has been shown to sensitize paclitaxel-resistant esophageal cancer cells in both *in vitro* and *in vivo* models (50). Similarly, the silencing of GUARDIN (a key regulator in the p53-mediated DNA damage response pathway) triggers apoptosis and decreases xenograft growth, thus suggesting that it may be a target for cancer treatment (22). Several promising compounds are currently under clinical investigation, including AURKA inhibitors (such as MK-5108, TAS-119, and KW-2449) and AURKB inhibitors (such as AZD1152, BI-831266, and chiauranib) (AZD1152, BI-831266, and chiauranib), which have been evaluated in clinical trials (NCT00543387 (51), NCT02448589 (52), NCT00346632 (53), NCT00338182 (54), and NCT02122809 (55), clinicaltrials.gov). Additionally, noncoding RNAs (e.g. miRNAs and lncRNAs) have been reported to be crucial regulators of gene expression and signaling pathways (56) in various malignancies. For example, exosomal miRNAs provide a dynamic perspective regarding the progression and therapeutic response (57), whereas DDIT4-AS1 is a potential therapeutic target for sensitizing TNBC to chemotherapy (58). In total, these developments underscore the potential of CIN-targeting strategies in precision oncology, thus offering new avenues for treating cancers that are resistant to conventional therapies.

In summary, our findings reveal a potential association between lncTRDMT1–5 and CIN-related phenotypes, EMT, and cell cycle modulation, suggesting a possible role in cancer progression. However, direct evidence of CIN - including chromosome mis-segregation events during mitosis, lagging chromosome frequency, micronucleus formation assay, and karyotypic or single-cell analyses - is required to firmly establish the role of lncTRDMT1–5 in CIN. Our findings not only establish a significant association between CIN and lncRNAs but also provide novel targeted intervention strategies for BC treatment. First, lncTRDMT1–5 may serve as a molecular biomarker, and its combination with genomic analysis techniques such as CMA could facilitate precise subtyping and prognostic evaluation; moreover, the incorporation of liquid biopsy approaches via the analysis of circulating cell-free DNA (cfDNA) from patients’ blood offers a noninvasive method for monitoring CIN dynamics. Second, the targeted inhibition of lncTRDMT1–5 or the modulation of its epigenetic modifications (e.g., H3K27ac) may exert antitumor effects by restoring p53 pathway function, reducing CIN levels, and reversing EMT phenotypes. Finally, the future development of lncTRDMT1-5-specific inhibitors (such as ASOs or small-molecule drugs) or the exploration of combination therapies with CIN-targeting agents such as AURKA inhibitors could offer new paradigms for personalized BC treatment.

## Data Availability

The original contributions presented in the study are included in the article/**Supplementary Material**. Further inquiries can be directed to the corresponding author.
