# Supplementary material for: LncTRDMT1–5 promotes chromosomal instability through MSRB3-mediated cell cycle and DNA damage in breast cancer
Source: Front Oncol. 2026 Aug 5;16:1899923. doi: 10.3389/fonc.2026.1899923 (PMC13485545; doi:10.3389/fonc.2026.1899923)
Supplement: Supplementary file 1 [file Table1.docx]

**Supplementary table 1**. Segments details (loss and gain) exist only in negative-control cells.

| **Microarray Nomenclature** | **CN State** | **Type** | **Size (kbp)** | **Genes** |
| --- | --- | --- | --- | --- |
| arr[GRCh38] 2p25.3(2887485_3120826)x1 | 1.00 | Loss | 233 | LINC01250 |
| arr[GRCh38] 2p25.2(5589075_5968787)x1 | 1.00 | Loss | 380 | LINC01248, SOX11, LINC01810, SILC1 |
| arr[GRCh38] 2p25.2p25.1(6064180_7466354)x1 | 1.00 | Loss | 1,402 | LINC01247, LINC01246, MIR7515HG, MIR7515, LINC00487, LOC100506274 |
| arr[GRCh38] 2p25.1(7897545_8733159)x1 | 1.00 | Loss | 836 | LOC101929551, LINC00298, LINC00299, LINC01814, ID2-AS1, ID2, KIDINS220 |
| arr[GRCh38] 2p24.3(12342314_12834277)x1 | 1.00 | Loss | 492 | MIR3681HG, TRIB2, MIR3125 |
| arr[GRCh38] 2p24.3(14225062_14452552)x1 | 1.00 | Loss | 227 | LINC00276 |
| arr[GRCh38] 2p24.3(14758223_15194984)x1 | 1.00 | Loss | 437 | NBAS |
| arr[GRCh38] 2p24.2(16557599_18065847)x1 | 1.00 | Loss | 1,508 | CYRIA, RAD51AP2, VSNL1, SMC6, GEN1, MSGN1, KCNS3 |
| arr[GRCh38] 2p24.1(22001474_23020054)x1 | 1.00 | Loss | 1,019 | LINC01884 |
| arr[GRCh38] 2p22.3(34350060_34851671)x1 | 1.00 | Loss | 502 | LINC01320 |
| arr[GRCh38] 2q31.2(178667260_178995770)x1 | 1.00 | Loss | 329 | TTN, LOC101927055, CCDC141 |
| arr[GRCh38] 2q31.3(181405155_181790414)x1 | 1.00 | Loss | 385 | ITGA4, CERKL, NEUROD1 |
| arr[GRCh38] 3p21.1(52715773_53129619)x1 | 1.00 | Loss | 414 | NEK4, ITIH1, ITIH3, ITIH4, ITIH4-AS1, MUSTN1, STIMATE-MUSTN1, STIMATE, MIR8064, SFMBT1, RFT1 |
| arr[GRCh38] 3p14.2(59374014_59919085)x1 | 1.00 | Loss | 545 | LOC339902, FHIT |
| arr[GRCh38] 4q13.3(70833086_71165745)x1 | 1.00 | Loss | 333 | GRSF1, MOB1B, DCK |
| arr[GRCh38] 6q12q13(69173970_69462997)x1 | 1.00 | Loss | 289 | ADGRB3 |
| arr[GRCh38] 8q13.2(68214813_68430645)x1 | 1.00 | Loss | 216 | PREX2, C8orf34-AS1, C8orf34 |
| arr[GRCh38] 10p14(7733092_7950604)x1 | 1.00 | Loss | 218 | ITIH2, KIN, ATP5F1C, TAF3 |
| arr[GRCh38] 10p13(13505709_14119507)x1 | 1.00 | Loss | 614 | BEND7, PRPF18, FRMD4A, LOC101928453 |
| arr[GRCh38] 10p12.31(20033916_20591458)x1 | 1.00 | Loss | 558 | PLXDC2, MIR4675 |
| arr[GRCh38] 10p11.23(29859880_30132025)x1 | 1.00 | Loss | 272 | JCAD |
| arr[GRCh38] 10p11.22(31453263_31813769)x1 | 1.00 | Loss | 361 | ZEB1, ARHGAP12 |
| arr[GRCh38] 10q26.3(132655193_132863454)x1 | 1.00 | Loss | 208 | INPP5A, NKX6-2, CFAP46 |
| arr[GRCh38] 11q23.1q23.2(112480812_113277582)x1 | 1.00 | Loss | 797 | LINC02763, LINC02764, LOC101928847, NCAM1, NCAM1-AS1 |
| arr[GRCh38] 11q23.3(118996277_119205129)x1 | 1.00 | Loss | 209 | CCDC84, RPL23AP64, RPS25, TRAPPC4, SLC37A4, HYOU1, VPS11, HMBS, H2AX, DPAGT1, C2CD2L, HINFP, ABCG4, NLRX1, PDZD3, CCDC153, SNORD150 |
| arr[GRCh38] 14q12(29800068_30019951)x1 | 1.00 | Loss | 220 | PRKD1 |
| arr[GRCh38] 14q23.3(66340271_67125555)x1 | 1.00 | Loss | 785 | CCDC196 |
| arr[GRCh38] 15q22.31(65397374_65627273)x1 | 1.00 | Loss | 230 | IGDCC4, DPP8, HACD3, INTS14, SLC24A1 |
| arr[GRCh38] 15q23(68404849_68662697)x1 | 1.00 | Loss | 258 | ITGA11, CORO2B |
| arr[GRCh38] 18q22.2(69481145_69697397)x1 | 1.00 | Loss | 216 | DOK6 |
| arr[GRCh38] 18q23(77256466_78125321)x1 | 1.00 | Loss | 869 | GALR1, LINC01029 |
| arr[GRCh38] 19q12(31256438_31572463)x1 | 1.00 | Loss | 316 | TSHZ3 |
| arr[GRCh38] 19q13.12(36705519_37778059)x1 | 1.00 | Loss | 1,073 | ZNF420, ZNF585A, ZNF585B, ZNF383, LINC01535, LOC284412, ZNF875, ZNF527, ZNF569, ZNF570, ZNF793-AS1, ZNF793, ZNF571-AS1, ZNF540, ZNF571, ZFP30, ZNF781, ZNF607, ZNF573 |
| arr[GRCh38] 19q13.41(51527236_52144907)x1 | 1.00 | Loss | 618 | SIGLEC6, ZNF175, LINC01530, SIGLEC5, SIGLEC14, SPACA6P-AS, SPACA6, MIR99B, MIRLET7E, MIR125A, HAS1, FPR1, FPR2, FPR3, ZNF577, ZNF649-AS1, ZNF649, ZNF613, ZNF350-AS1, ZNF350, ZNF615, ZNF614, ZNF432, ZNF841, ZNF616 |
| arr[GRCh38] 20p13(81022_308312)x1 | 1.00 | Loss | 227 | DEFB125, DEFB126, DEFB127, DEFB128, DEFB129, DEFB132, C20orf96, ZCCHC3 |
| arr[GRCh38] 20p13(364102_721315)x1 | 1.00 | Loss | 357 | TRIB3, RBCK1, TBC1D20, CSNK2A1, TCF15, SRXN1, SCRT2 |
| arr[GRCh38] 21q21.1(17894495_18150215)x1 | 1.00 | Loss | 256 | CHODL |
| arr[GRCh38] 1p32.1(58846669_59375452)x3 | 3.00 | Gain | 529 | LINC01135, LINC01358, HSD52, FGGY |
| arr[GRCh38] 1p31.1(72307577_73316465)x3 | 3.00 | Gain | 1,009 | LINC01360 |
| arr[GRCh38] 1p21.3p21.2(98747627_100261910)x3 | 3.00 | Gain | 1,514 | SNX7, PLPPR5, LOC100129620, PLPPR4, LINC01708, MIR548N, PALMD, FRRS1, AGL, SLC35A3, MFSD14A, SASS6, TRMT13, LRRC39, DBT |
| arr[GRCh38] 1p13.3(107380943_108333074)x3 | 3.00 | Gain | 952 | NTNG1, VAV3, MIR7852, VAV3-AS1, SLC25A24, NBPF4 |
| arr[GRCh38] 1p13.3(108682178_108988807)x3 | 3.00 | Gain | 307 | PRPF38B, FNDC7, STXBP3, AKNAD1, SPATA42, GPSM2, CLCC1, WDR47 |
| arr[GRCh38] 1p13.2(111569430_111803865)x3 | 3.00 | Gain | 234 | RAP1A, LINC01160, LOC107985184, INKA2, INKA2-AS1, LOC101928718, DDX20, KCND3 |
| arr[GRCh38] 2q11.2(97851062_98111300)x3 | 3.00 | Gain | 260 | TMEM131, VWA3B |
| arr[GRCh38] 2q11.2(99061732_99571126)x3 | 3.00 | Gain | 509 | TSGA10, C2orf15, LIPT1, MITD1, MRPL30, LYG2, LYG1, TXNDC9, EIF5B, REV1, AFF3 |
| arr[GRCh38] 2q11.2q12.1(100296001_102207239)x3 | 3.00 | Gain | 1,911 | TBC1D8-AS1, CNOT11, SNORD89, RNF149, MIR5696, CREG2, RFX8, MAP4K4, LINC01127, IL1R2, IL1R1, IL1RL2 |
| arr[GRCh38] 2q12.1(103618761_104762172)x3 | 3.00 | Gain | 1,143 | LOC100287010, LINC01831, LINC01102, LINC01103, LINC01114 |
| arr[GRCh38] 2q12.3(107220046_107692720)x3 | 3.00 | Gain | 473 | MIR548AU, LINC01886 |
| arr[GRCh38] 2q13(109740565_111933232)x3 | 3.00 | Gain | 2,193 | RGPD5, RGPD6, RGPD8, LIMS4, LIMS3-LOC440895, LIMS3, LOC100288570, LOC440895, LINC01123, MIR4267, MALL, MIR4436B1, MIR4436B2, NPHP1, MTLN, LOC100507334, LINC01106, LOC105373553, BUB1, SNORD132, ACOXL, ACOXL-AS1, BCL2L11, MIR4435-2HG, MIR4435-1, MIR4435-2, ANAPC1, MIR4771-1, MIR4771-2, MERTK |
| arr[GRCh38] 2q14.1(112472128_112723309)x3 | 3.00 | Gain | 251 | TTL, LOC105373562, POLR1B, CHCHD5, FLJ42351, SLC20A1, NT5DC4 |
| arr[GRCh38] 2q14.1(115139780_116511637)x3 | 3.00 | Gain | 1,372 | DPP10, DPP10-AS1 |
| arr[GRCh38] 2q14.3(124381969_125326867)x3 | 3.00 | Gain | 945 | CNTNAP5 |
| arr[GRCh38] 2q14.3(127125542_128069416)x3 | 3.00 | Gain | 944 | CYP27C1, ERCC3, MAP3K2, PROC, MIR4783, IWS1, MYO7B, LOC105373609, LIMS2, GPR17, WDR33, SFT2D3, POLR2D, AMMECR1L, SAP130 |
| arr[GRCh38] 3q27.3q28(187862353_188270501)x3 | 3.00 | Gain | 408 | LINC01991, LPP-AS2, LPP, FLJ42393 |
| arr[GRCh38] 4q32.1(158219607_158478430)x3 | 3.00 | Gain | 259 | TMEM144 |
| arr[GRCh38] 5q13.2(73593748_73958016)x3 | 3.00 | Gain | 364 | ARHGEF28 |
| arr[GRCh38] 5q13.3(76596898_77459556)x3 | 3.00 | Gain | 863 | IQGAP2, LOC101929109, F2RL2, NCRUPAR, F2R, F2RL1, S100Z, CRHBP, AGGF1, ZBED3, SNORA47, ZBED3-AS1, PDE8B, WDR41 |
| arr[GRCh38] 5q14.3(84098136_85202646)x3 | 3.00 | Gain | 1,105 | EDIL3 |
| arr[GRCh38] 5q31.1(135068276_135468962)x3 | 3.00 | Gain | 401 | C5orf66, C5orf66-AS2, MACROH2A1, DCANP1, TIFAB |
| arr[GRCh38] 5q32(146593807_147126924)x3 | 3.00 | Gain | 533 | PPP2R2B, PPP2R2B-IT1 |
| arr[GRCh38] 5q34(162223147_168620042)x3 | 3.00 | Gain | 6,397 | LINC02143, LOC102546299, LINC01947, TENM2, LOC101927908, MIR12125, CTB-178M22.2, WWC1, RARS1, FBLL1, PANK3, MIR103A1, MIR103B1 |
| arr[GRCh38] 5q35.1(170894580_171225909)x3 | 3.00 | Gain | 331 | RANBP17 |
| arr[GRCh38] 6p25.1p24.3(6356249_8463748)x3 | 3.00 | Gain | 2,108 | LY86-AS1, LY86, RREB1, BMP6, BLOC1S5-TXNDC5, TXNDC5, PIP5K1P1, BLOC1S5, EEF1E1-BLOC1S5, EEF1E1, SCARNA27, |
| arr[GRCh38] 6p24.3(8738749_10495003)x3 | 3.00 | Gain | 1,756 | LOC100506207, TFAP2A, TFAP2A-AS2, TFAP2A-AS1, LINC00518, MIR5689HG, MIR5689 |
| arr[GRCh38] 6p24.1p23(11907482_14229471)x3 | 3.00 | Gain | 2,322 | LOC101928253, HIVEP1, EDN1, LINC02530, PHACTR1, TBC1D7-LOC100130357, LOC100130357, TBC1D7, GFOD1, SIRT5, NOL7, RANBP9, MCUR1 |
| arr[GRCh38] 6p22.3(20479811_22083036)x3 | 3.00 | Gain | 1,603 | E2F3, CDKAL1, LINC00581, SOX4, CASC15 |
| arr[GRCh38] 6p22.1(27774262_28990572)x3 | 3.00 | Gain | 1,216 | H2BC13, H2AC13, H3C10, H2AC14, H2BC14, H4C11, H4C12, H2AC15, H2BC15, H2AC16, H1-5, H3C11, H4C13, H3C12, H2AC17, H2BC17, OR2B2, OR2B6, OR1F12, ZNF165, ZSCAN12P1, ZSCAN16-AS1, ZSCAN16, ZKSCAN8, ZNF192P1, TOB2P1, ZSCAN9, ZKSCAN4, NKAPL, ZSCAN26, PGBD1, ZSCAN31, ZKSCAN3, ZSCAN12, ZSCAN23, GPX6, GPX5, ZBED9, LINC00533, LINC01623, HCG14, TRIM27, LINC01556, HCG15 |
| arr[GRCh38] 6p21.33(30628358_30889765)x3 | 3.00 | Gain | 261 | ATAT1, C6orf136, DHX16, PPP1R18, NRM, MDC1, MDC1-AS1, TUBB, FLOT1, IER3-AS1, IER3, HCG20, LINC00243, LINC02570, DDR1 |
| arr[GRCh38] 7p21.2(14108246_15015179)x3 | 3.00 | Gain | 907 | DGKB |
| arr[GRCh38] 7q11.21(65017860_65992731)x3 | 3.00 | Gain | 975 | CCT6P3, SNORA22C, SNORA15B-1, SNORA15B-2, INTS4P1, ZNF92, LOC101929322, LOC441242, INTS4P2, CCT6P1, SNORA22, VKORC1L1, GUSB |
| arr[GRCh38] 7q21.12q21.13(88311033_88813917)x3 | 3.00 | Gain | 503 | ZNF804B, TEX47 |
| arr[GRCh38] 7q21.13(89699531_90726803)x3 | 3.00 | Gain | 1,027 | STEAP2-AS1, DPY19L2P4, STEAP1, STEAP2, CFAP69, LOC101927446, GTPBP10, LOC101409256, CLDN12, LOC102723899, CDK14 |
| arr[GRCh38] 7q21.2(91776537_92008073)x3 | 3.00 | Gain | 232 | MTERF1, AKAP9 |
| arr[GRCh38] 7q22.1(103186195_103850092)x3 | 3.00 | Gain | 664 | DPY19L2P2, PMPCB, DNAJC2, PSMC2, SLC26A5, LOC101927870, RELN |
| arr[GRCh38] 7q22.3(105808703_106143867)x3 | 3.00 | Gain | 335 | ATXN7L1, CDHR3, SYPL1 |
| arr[GRCh38] 7q31.1(108041845_108870123)x3 | 3.00 | Gain | 828 | LAMB4, NRCAM, PNPLA8, THAP5, DNAJB9 |
| arr[GRCh38] 7q31.1(109439458_110596729)x3 | 3.00 | Gain | 1,157 | EIF3IP1 |
| arr[GRCh38] 7q31.1(112925852_113229936)x3 | 3.00 | Gain | 304 | BMT2, HRAT17, GPR85, SMIM30 |
| arr[GRCh38] 7q31.2q31.31(116794554_117762249)x3 | 3.00 | Gain | 968 | MET, CAPZA2, ST7-AS1, ST7, ST7-OT4, MIR6132, ST7-AS2, ST7-OT3, WNT2, ASZ1, CFTR, CFTR-AS1, CTTNBP2 |
| arr[GRCh38] 7q31.32q31.33(123825339_126128033)x3 | 3.00 | Gain | 2,303 | HYAL4, SPAM1, LOC105375483, TMEM229A, LOC101928211, GPR37, C7orf77, POT1, POT1-AS1, LOC101928283, LOC101928254 |
| arr[GRCh38] 7q35(143441563_144533302)x3 | 3.00 | Gain | 1,092 | TCAF2, EPHA1-AS1, TAS2R60, TAS2R41, OR10AC1, CTAGE15, TCAF2P1, CTAGE6, LOC154761, TCAF1, OR2F2, OR2F1, OR6B1, OR2A5, OR2A25, OR2A12, OR2A2, OR2A14, CTAGE8, CTAGE4, ARHGEF35, ARHGEF35-AS1, OR2A1-AS1, OR2A1, OR2A42, OR2A9P, OR2A20P, OR2A7, ARHGEF34P, ARHGEF5, NOBOX, TPK1 |
| arr[GRCh38] 8q22.3(101177829_101446777)x3 | 3.00 | Gain | 269 | ZNF706, NACA4P |
| arr[GRCh38] 8q24.21(128105690_129341435)x3 | 3.00 | Gain | 1,236 | MIR1208, LINC00824, LINC00976, LINC00977 |
| arr[GRCh38] 8q24.3(140199992_140755651)x3 | 3.00 | Gain | 556 | TRAPPC9, CHRAC1, AGO2, PTK2, MIR151A |
| arr[GRCh38] 8q24.3(142731523_143143387)x3 | 3.00 | Gain | 412 | THEM6, SLURP1, LYPD2, SLURP2, LYNX1-SLURP2, LYNX1, LY6D, GML, CYP11B1, CYP11B2, LY6E-DT, CDC42P3, LY6E, C8orf31, MIR1302-7, LY6L |
| arr[GRCh38] 9p22.2p22.1(16911093_19224327)x3 | 3.00 | Gain | 2,313 | CNTLN, SH3GL2, ADAMTSL1, MIR3152, |
| arr[GRCh38] 9q21.13(74603429_75085169)x3 | 3.00 | Gain | 482 | RORB, TRPM6, C9orf40, CARNMT1-AS1, CARNMT1, NMRK1 |
| arr[GRCh38] 9q21.33(85219757_86927595)x3 | 3.00 | Gain | 1,708 | AGTPBP1, LOC389765, NAA35, GOLM1, LOC101927623, C9orf153, ISCA1, TUT7 |
| arr[GRCh38] 10q22.3(77904669_78127699)x3 | 3.00 | Gain | 223 | DLG5, DLG5-AS1, POLR3A, RPS24 |
| arr[GRCh38] 10q23.2(87118873_87540596)x3 | 3.00 | Gain | 422 | SHLD2, NUTM2A, NUTM2A-AS1, LINC00863, NUTM2D, FAM245A, MIR4678, MINPP1 |
| arr[GRCh38] 11p15.1(20745445_21368965)x3 | 3.00 | Gain | 624 | NELL1 |
| arr[GRCh38] 11p13(35739191_36299905)x3 | 3.00 | Gain | 561 | TRIM44, LDLRAD3, MIR3973, LOC101928510, COMMD9, PRR5L |
| arr[GRCh38] 11q13.1q13.2(65526666_66828177)x3 | 3.00 | Gain | 1,302 | SCYL1, LTBP3, ZNRD2-AS1, ZNRD2, FAM89B, EHBP1L1, KCNK7, MAP3K11, PCNX3, MIR4690, SIPA1, MIR4489, RELA, KAT5, RNASEH2C, AP5B1, OVOL1, OVOL1-AS1, SNX32, CFL1, MUS81, EFEMP2, CTSW, FIBP, CCDC85B, FOSL1, C11orf68, DRAP1, TSGA10IP, SART1, EIF1AD, BANF1, CST6, CATSPER1, GAL3ST3, SF3B2, SNORD13F, PACS1, KLC2, RAB1B, CNIH2, YIF1A, TMEM151A, CD248, RIN1, BRMS1, B4GAT1, LOC102724064, SLC29A2, NPAS4, MRPL11, PELI3, LOC101928069, DPP3, BBS1, ZDHHC24, ACTN3, CTSF, CCDC87, CCS, RBM14-RBM4, RBM14, RBM4, RBM4B, SPTBN2, C11orf80 |
| arr[GRCh38] 12p12.3p12.2(15004981_20941093)x3 | 3.00 | Gain | 5,936 | LINC01489, LMO3, SKP1P2, LINC02378, MIR3974, RERGL, PIK3C2G, PLCZ1, CAPZA3, SLCO1B3, SLCO1B3-SLCO1B7 |
| arr[GRCh38] 12q21.31q21.33(84652935_88726446)x3 | 3.00 | Gain | 4,074 | SLC6A15, TSPAN19, LRRIQ1, ALX1, RASSF9, NTS, MGAT4C, MIR3059, KITLG |
| arr[GRCh38] 12q24.23(118239135_120109823)x3 | 3.00 | Gain | 1,871 | TAOK3, SUDS3, LINC02423, LINC02439, SRRM4, LOC105370024, HSPB8, LINC00934, |
| arr[GRCh38] 12q24.31(121522075_122813985)x3 | 3.00 | Gain | 1,292 | KDM2B, MIR548AQ, ORAI1, MORN3, TMEM120B, RHOF, LINC01089, SETD1B, HPD, PSMD9, WDR66, BCL7A, LOC100506691, MLXIP, LRRC43, IL31, B3GNT4, DIABLO, LOC101593348, VPS33A, CLIP1, CLIP1-AS1, ZCCHC8, RSRC2, KNTC1, MIR9902-1, MIR9902-2, HCAR2, HCAR3, HCAR1, DENR, CCDC62 |
| arr[GRCh38] 12q24.31(123801765_124230251)x3 | 3.00 | Gain | 428 | DNAH10, CCDC92, ZNF664, ZNF664-RFLNA |
| arr[GRCh38] 15q21.1(47934461_48590260)x3 | 3.00 | Gain | 656 | SLC24A5, MYEF2, CTXN2, SLC12A1, DUT, FBN1 |
| arr[GRCh38] 15q21.2q21.3(52472368_52908793)x3 | 3.00 | Gain | 436 | MYO5A, ARPP19, FAM214A, ONECUT1 |
| arr[GRCh38] 15q21.3(54252813_55738558)x3 | 3.00 | Gain | 1,486 | UNC13C, LOC105370829, RSL24D1, RAB27A, PIGBOS1, PIGB, CCPG1, DNAAF4-CCPG1, MIR628, C15orf65, DNAAF4, PYGO1, PRTG |
| arr[GRCh38] 15q21.3(57003112_57235368)x3 | 3.00 | Gain | 232 | TCF12 |
| arr[GRCh38] 15q21.3q22.2(57711130_59003803)x3 | 3.00 | Gain | 1,293 | GCOM1, POLR2M, ALDH1A2, LOC283665, AQP9, LIPC, LIPC-AS1, ADAM10, HSP90AB4P, LOC101928725, MINDY2, SLTM, RNF111 |
| arr[GRCh38] 15q25.3(84829520_85671870)x3 | 3.00 | Gain | 842 | ALPK3, SLC28A1, PDE8A, LOC101929479, GOLGA6L3, MIR7706, AKAP13 |
| arr[GRCh38] 16p13.3(35881_243643)x3 | 3.00 | Gain | 208 | POLR3K, SNRNP25, RHBDF1, MPG, NPRL3, HBZ, HBM, HBA2, HBA1, HBQ1, LUC7L, FAM234A |
| arr[GRCh38] 16p13.11(15698180_16008865)x3 | 3.00 | Gain | 311 | NDE1, MYH11, CEP20, ABCC1 |
| arr[GRCh38] 16p11.2(28737296_29578353)x3 | 3.00 | Gain | 841 | NPIPB9, ATXN2L, TUFM, MIR4721, SH2B1, ATP2A1, ATP2A1-AS1, RABEP2, CD19, NFATC2IP, MIR4517, SPNS1, LAT, RRN3P2, SNX29P2, NPIPB11, SMG1P6, BOLA2-SMG1P6, LOC606724, BOLA2, BOLA2B, SLX1B, SLX1A, SLX1A-SULT1A3, SLX1B-SULT1A4, SULT1A3, SULT1A4, LOC388242, LOC613038, NPIPB12, SMG1P2 |
| arr[GRCh38] 16q22.1q22.2(70293139_71320340)x3 | 3.00 | Gain | 1,027 | DDX19B, LOC100506083, DDX19A, ST3GAL2, FCSK, COG4, SF3B3, SNORD111B, SNORD111, IL34, MTSS2, VAC14, VAC14-AS1, HYDIN, CMTR2 |
| arr[GRCh38] 16q23.2q23.3(81116478_81880360)x3 | 3.00 | Gain | 764 | PKD1L2, BCO1, GAN, MIR4720, CMIP, MIR7854, MIR6504, LOC100129617, PLCG2 |
| arr[GRCh38] 17q21.31(44855541_45174864)x3 | 3.00 | Gain | 319 | EFTUD2, CCDC103, GFAP, FAM187A, KIF18B, MIR6783, C1QL1, DCAKD, NMT1, PLCD3, MIR6784, ACBD4, HEXIM1, HEXIM2, LOC105371795 |
| arr[GRCh38] 17q21.31(45408425_45765128)x3 | 3.00 | Gain | 357 | ARHGAP27, PLEKHM1, MIR4315-1, MIR4315-2, LRRC37A4P, ARL17A, ARL17B, MAPK8IP1P2, LINC02210, LINC02210-CRHR1 |
| arr[GRCh38] 17q21.33(49934449_50422093)x3 | 3.00 | Gain | 488 | DLX4, DLX3, PICART1, ITGA3, PDK2, SAMD14, PPP1R9B, SGCA, H1-9P, COL1A1, LINC01969, TMEM92, TMEM92-AS1, XYLT2, MRPL27, EME1, LRRC59 |
| arr[GRCh38] 17q21.33(51141436_51532414)x3 | 3.00 | Gain | 391 | NME1, NME1-NME2, NME2, MBTD1, UTP18, LINC02071, LINC02072, LINC02073 |
| arr[GRCh38] 17q23.1q23.2(59909736_61689586)x3 | 3.00 | Gain | 1,780 | RPS6KB1, RNFT1, TBC1D3P1-DHX40P1, RNFT1-DT, HEATR6, MIR4737, LOC105371849, WFDC21P, LOC653653, CA4, USP32, SCARNA20, C17orf64, APPBP2, LINC01999, PPM1D, BCAS3, LOC101927855, TBX2-AS1, TBX2, LINC02875, TBX4, NACA2, BRIP1 |
| arr[GRCh38] 17q24.3(69849659_71430907)x3 | 3.00 | Gain | 1,581 | LINC01483, LINC01497, LINC01028, KCNJ16, KCNJ2-AS1, KCNJ2, CASC17 |
| arr[GRCh38] 17q25.1(75804109_76459770)x3 | 3.00 | Gain | 656 | UNK, UNC13D, WBP2, TRIM47, TRIM65, MRPL38, FBF1, ACOX1, TEN1-CDK3, TEN1, CDK3, EVPL, SRP68, GALR2, ZACN, EXOC7, MIR6868, FOXJ1, RNF157-AS1, RNF157, UBALD2, QRICH2, PRPSAP1, SPHK1, UBE2O, AANAT |
| arr[GRCh38] 17q25.3(77970512_79159574)x3 | 3.00 | Gain | 1,189 | SOCS3, LOC101928674, PGS1, DNAH17, DNAH17-AS1, SCAT1, CYTH1, USP36, TIMP2, CEP295NL, LGALS3BP, CANT1, C1QTNF1-AS1, C1QTNF1, ENGASE, RBFOX3 |
| arr[GRCh38] 17q25.3(80125169_80977353)x3 | 3.00 | Gain | 852 | EIF4A3, CARD14, SGSH, SLC26A11, RNF213, RNF213-AS1, ENDOV, MIR4730, NPTX1, RPTOR, LOC101928855, LOC400627 |
| arr[GRCh38] 18p11.23p11.22(7448774_8554835)x3 | 3.00 | Gain | 1,106 | LOC112577592, PTPRM, LOC100192426 |
